# Acupuncture at Gastric Back-Shu and Front-Mu Acupoints Enhances Gastric Motility via the Inhibition of the Glutamatergic System in the Hippocampus

**DOI:** 10.1155/2020/3524641

**Published:** 2020-03-10

**Authors:** Hao Wang, Wen-Jian Liu, Meng-Jie Hu, Meng-Ting Zhang, Guo-Ming Shen

**Affiliations:** ^1^Institute of Integrated Chinese and Western Medicine, Anhui University of Traditional Chinese Medicine, Hefei 230012, Anhui Province, China; ^2^Department of Thoracic Surgery, The First Affiliated Hospital of Anhui Medical University, Hefei 230032, Anhui Province, China; ^3^College of Integrated Chinese and Western Medicine, Anhui University of Traditional Chinese Medicine, Hefei 230012, Anhui Province, China

## Abstract

Acupuncture strongly alleviates gastrointestinal symptoms and especially promotes gastrointestinal motility. However, the mechanism underlying these processes is poorly understood. This study was designed to examine the effect of electroacupuncture (EA) at gastric back-shu (BL21) and front-mu (RN12) acupoints on gastric motility in functional dyspepsia (FD) rats and to investigate the mechanisms of its effects on the glutamatergic system in the hippocampus. We found that EA at RN12 or BL21 enhanced gastric motility in FD rats, whereas EA at the combination of RN12 and BL21 showed an additional effect. Microdialysis combined with HPLC showed that EA reduced the glutamate content in the hippocampus, and the NMDAR-NO-cGMP signalling pathway was downregulated, as determined by Western blot assays, in FD rats. In addition, we found that decreased gastric motility was significantly restored by the hippocampal infusion of an NMDAR, nNOS, or sGC antagonist. Interestingly, EA had no further effects on gastric motility in the presence of these antagonists in FD rats. Taken together, these results suggest that the hippocampal glutamatergic system is involved in the regulation of gastric motility by EA at RN12 and BL21.

## 1. Introduction

Functional dyspepsia (FD) is a common functional gastrointestinal (GI) disease, and the global incidence of FD is 11.5%–29.2% [1, 2]. There are various options for treating FD, such as diet, prokinetic agents, acid suppression, fundic relaxers, tricyclic antidepressants, and psychological therapy, but the treatments are unsatisfactory [3].

Acupuncture has long been used in China to treat FD, and it has been used in some Western countries as an optional treatment for gastrointestinal diseases due to its high efficacy and safety [4–6]. Studies have shown that RN12, ST36, ST37, PC6, ST25, and BL21 are principal acupoints for the treatment of gastrointestinal diseases [7]. Notably, specific combinations of acupoints may have additional effects. For example, the combination of RN12 and BL21 elicits an additional effect on intragastric pressure [8, 9]. However, many studies on the mechanism of acupuncture have focused on a single acupoint, and the mechanism underlying the additional effects of acupoint compatibility is poorly understood. In this study, we investigated the effect of electroacupuncture (EA) at a combination of RN12 and BL21 on gastric motility in FD rats and the mechanisms underlying these effects.

Accumulating evidence has suggested that there are important links between the central nervous system and the stomach that have significant effects on gastric function and that the stomach also affects the brain [10, 11]. Recently, studies on the mechanism by which acupuncture regulates gastrointestinal diseases have also focused on areas of the central nervous system, such as the dorsal nucleus of the vagus nerve, locus coeruleus, paraventricular nucleus, amygdala, and raphe nucleus, and the limbic system was found to have the strongest association [12]. For example, one study showed that the hippocampus participates in the effect of electroacupuncture and enhances the intestinal propulsive rate in FD rats [13]. Another study indicated that gastric nutrient infusion evokes greater activation in the hippocampus [14]. These findings reveal that the hippocampus may play a role in the regulation of gastrointestinal function. Whether and how the hippocampus is involved in the improvement of FD by electroacupuncture at RN12 and BL21 is unknown.

It has been shown that central glutamatergic neurons regulate phase II contractions of migrating motor contractions [15]. Some studies have indicated the existence of many gastric dilatation-sensitive neurons in the hippocampus [16], but the types of these neurons are unclear. In addition, glutamatergic signalling in the dorsal motor nucleus of the vagus (DMV) via the activation of N-methyl-d-aspartate receptors (NMDAR) increases gastric motility [17]; NMDAR is widely distributed in the hippocampus [18]. The stimulation of NMDARs results in calcium influx, the activation of neuronal nitric oxide synthase (nNOS), and an increase in the content of nitric oxide (NO) [19, 20]. NO plays an important role in regulating gastrointestinal motility, and most of the physiological processes of NO result from its activation of soluble guanylate cyclase (sGC), an enzyme that catalyses the generation of cyclic guanosine monophosphate (cGMP) [21–23]. These findings suggest that glutamate and the NMDAR-NO-cGMP pathway are involved in regulating gastric motility. However, little is known about their role in the regulation of gastric motility by EA at BL21 and RN12. In this study, we investigated whether EA at BL21 and RN12 improves gastric motility via regulating glutamate and the NMDAR-NO-cGMP pathway in the hippocampus.

## 2. Materials and Methods

### 2.1. Animals and Experimental Design

**Figure 1:**
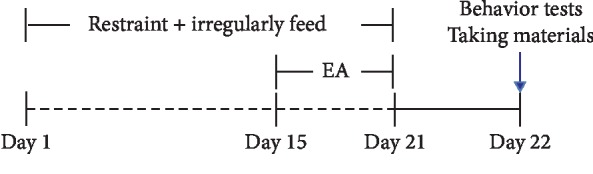
The experimental protocol.

Adult male Sprague-Dawley rats (SD, 250–300 g) were obtained from Qinglongshan Animal Breeding Farm (Nanjing, Jiangsu, China) and housed under controlled conditions (22∼24°C, lights on from 6:00 AM to 6:00 PM) with free access to food and water. All of the procedures were approved by the Anhui University of Traditional Chinese Medicine Animal guidelines for care and use of experimental animals. The functional dyspepsia (FD) model was established by restraining rats in homemade well-ventilated cylindrical tubes (150 mm in length, 60 mm in diameter) for 60 min once per day and irregularly feeding them (fed on one day, fasting for one day) for 21 days [24]. After restraining and irregularly feeding for 21 days, the rats showed diet reduction, activities being decreased, feces rarefaction, and hair scorch. Then, we recorded the gastric motility and found that hypomotility appeared in the model rats, which indicates that the FD model was successful. The EA stimulation was carried out on the 15^th^ day of model building. The experimental protocol is shown in Figure 1.

The study includes three experiments. In experiment 1, the rats were randomly assigned to five groups (*n* = 12/group): control group, FD group (restraint + irregularly fed), RN12 group (FD rats with EA at RN12), BL21 group (FD rats with EA at BL21), and RN12 + BL21 group (FD rats with EA at RN12 plus BL21). When the food intake was detected (*n* = 6), the rats were perfused with saline and PFA, and then the brain was removed to determine the expression of NR1 by immunostaining. And when the gastric motility was recorded (*n* = 6), the hippocampi were removed to determine the concentration of glutamate by colorimetric assay and the expression of NR1, nNOS, and sGC by Western blotting. In experiment 2, the groups were the same as in experiment 1 (*n* = 6); in this experiment, we examined the concentration of glutamate in the hippocampus by microdialysis combined with HPLC. In experiment 3, the rats were randomly divided into seven groups to record the gastric motility (*n* = 6): FD group (FD rats with injection of normal saline into hippocampi), MK-801 group (FD rats with injection of MK-801 into hippocampi), MK-801 + EA group (FD rats with injection of MK-801 plus EA at RN12 + BL21), l-NAME group (FD rats with injection of L-NAME into hippocampi), l-NAME + EA group (FD rats with injection of L-NAME plus EA at RN12 + BL21), MB group (FD rats with injection of MB into hippocampi), and MB + EA group (FD rats with injection of MB plus EA at RN12 + BL21).

### 2.2. Electroacupuncture (EA) Stimulation

The location of the acupoints was consistent with those used in our previous study [8]. RN12 is located on the midline of the upper abdomen, 20 mm above the umbilicus, and BL21 is located 5 mm on side of the twelfth thoracic vertebra. The EA process was conducted with needles (0.35 mm ∗ 13 mm, Hua Tuo, Suzhou, China) and an EA instrument (SDZ-IV, Hua Tuo, Suzhou, China) for 20 min each day for 7 days with the following parameters: frequency of 2 Hz and current intensity of 2 mA.

### 2.3. Food Intake and Gastric Motility Measurements

To evaluate the FD model and the effect of EA, we recorded the food intake and gastric motility. Rats that had been fasted overnight were allowed to freely consume water and a preweighed amount of solid food for 30 min, After that, the water and food were removed, and the amount of food was measured again. Food intake was calculated by this formula: food intake = preweighed amount − remaining amount.

The method of recording gastric motility was similar to that used in previous studies [25]. Firstly, we used pentobarbital (50 mg/kg, i.p.) to anaesthetize the rats and then opened the abdomen to expose the gastric antrum. A strain gauge with a gastrointestinal pressure sensor (Xinhang Xingye Technology and Trade Co., Ltd. Beijing, China) was embedded into the subserosal layer of the gastric antrum, and a Powerlab 8/30 biological signal acquisition system and LabChart analysis software (AD Instruments International Trading Co., Ltd. Shanghai, China) were used to record the waves of gastric motility and to analyse the gastric motility amplitude and the gastric motility index. The motility index was defined as the product of amplitude and frequency within 2 minutes [26].

### 2.4. Microinjection of the Hippocampus

The rats were anaesthetized with pentobarbital (50 mg/kg, i.p.) and then fixed on a brain stereotaxic instrument (Stoelting, Wood Dale, IL, USA). According to the coordinates of Paxinos and Watson (mediolateral: ±4.5 mm, anteroposterior: −5.3 mm, dorsoventral: −3.3 mm), 1 *μ*L normal saline (NS), an NMDAR antagonist (MK-801; 1 *μ*L, 0.5 *μ*g/*μ*L), and an nNOS inhibitor (L-NAME; 100 nL; 1 mol/L) or an sGC inhibitor (MB; 100 nL; 100 mmol/L) (all provided by MedChem Express Company) were injected into the bilateral hippocampi with a microsyringe (Anting Scientific Instrument, Shanghai, China).

### 2.5. Colorimetric Assay of Glutamate

To examine the concentration of glutamate in the hippocampi, pentobarbital (50 mg/kg, i.p.) was used to anaesthetize the rats, and the bilateral hippocampi were removed and stored at −80°C. According to the instructions of the Glutamate Assay Kit (MAK004, Sigma-Aldrich), the glutamate standard was first used to generate linear standard curves (*R*^2^ = 0.9989, the linear range was between 2 and 10 nmol). The samples were homogenized and diluted at a constant ratio with Glutamate Assay Buffer to ensure that the readings are within the linear range of the standard curve and then 30 *μ*L samples were collected and mixed with various reagents according to the instructions of the kit. The absorbance was detected at 340 nm. The concentration of glutamate (*μ*g/ml or ng/*μ*L) was calculated based on the formula: *C* = *S*_a_/*S*_v_, (Sa: amount of glutamate in sample (nmol) from standard curve; S_v_: sample volume (*μ*L) added to the wells; glutamate molecular weight: 147.3 ng/nmol).

### 2.6. *In Vivo* Microdialysis Combined with HPLC Test of Glutamate

Rats were anaesthetized with pentobarbital (50 mg/kg, i.p.) and then fixed to a brain stereotaxic instrument (Stoelting, Wood Dale, IL, USA). A hole was drilled for a cannula (CMA12, CMA, USA), and a cannula was stereotactically implanted into the right hippocampus (mediolateral: −4.5 mm; anteroposterior: −5.3 mm; dorsoventral: −3.3 mm). After the surgery, the rats were individually housed and allowed to recover for at least three days. A 4 mm CMA12 probe was placed into the cannula, and Ringer's solution (145 mM NaCl, 3 mM KCl, 1.3 mM CaCl_2_·2H_2_O) was perfused through the probe at a flow rate of 2 *μ*L/min with a CMA402 syringe pump. Fifteen tubes of microdialysate samples (10 min/tube) were collected from each rat.

The collected samples were stored at −80°C. The concentration of glutamate was analysed by HPLC (ANTEC, Netherlands). The mobile phase component was 19.1968 g of NaH_2_PO_4_ and 400 mL of methyl alcohol, pH = 3.48, in 2 L. A total of 20 *μ*L of each sample was mixed with 5 *μ*L of OPA (25 mg OPA, 250 *μ*L methyl alcohol, 250 *μ*L 1 mol/L Na_2_SO_3_, and 4.5 mL boric acid) for 3 min, and the compounds were separated on a 1 mm × 50 mm column (ALF-105, ANTEC). An online analysis system (ANTEC Leyden), which consisted of a DECADE II electrochemical detector and VT-3 electrochemical flow cells, was used for detection [27]. The data were analysed using Clarity software (ANTEC, Netherlands) based on standard samples.

### 2.7. Western Blotting Analysis of NR1, nNOS, and sGC

Total protein was extracted using a protein extraction kit (Biyuntian Biotech Corp., Shanghai, China), and the concentrations of the samples were detected by the bicinchoninic acid (BCA) method. An equal amount of protein from each sample was loaded, separated, transferred to nitrocellulose (NC) filter membranes, and then blocked at room temperature for 2 h. Primary antibodies (NR1, 1 : 2000; nNOS, 1 : 2000; sGC, 1 : 5000) (Abcam, USA) diluted in antidilution solution were added to the membranes and incubated at 4°C overnight. The membranes were then incubated with a secondary antibody (Horseradish peroxidase-labeled goat anti-rabbit IgG) for 2 h at room temperature. The protein bands were detected by an FCM gel imaging system (ProteinSimple, USA). The antibodies mentioned above were all purchased from Abochorage Shanghai Trading Co., Ltd. The band intensities were analysed using ImageJ software. Three rats from each group were used.

### 2.8. Immunohistochemical Analysis of NR1

Rats were anaesthetized with pentobarbital (50 mg/kg, i.p.) and perfused with 0.9% saline and then with 4% paraformaldehyde (PFA). The brains were dissected and fixed with 4% PFA for 24 hours at 4°C and then transferred to 20% sucrose for 10 hours and 30% sucrose for 10 hours. After that, the brains were embedded in OCT, and the brains were sliced into coronal sections (40 *μ*m) and incubated overnight at 4°C with a primary antibody (NR1, 1 : 500, Abcam, USA). Then, the sections were incubated with a secondary antibody (sheep anti-rabbit IgG, 1 : 500, Abcam, USA) for 2 hours. Then, the sections were observed under an electron microscope (Olympus, Japanese), and Image-Pro Plus 6.0 software was used for quantification.

### 2.9. Statistical Analysis

All experimental values are expressed as the means ± SD. Comparisons among multiple groups were analysed using one-way ANOVA, and comparisons between any two groups among multiple groups were analysed using Tukey's method. A *P* value <0.05 was considered statistically significant.

## 3. Results

### 3.1. The Effect of EA at RN12 and BL21 on Gastric Motility

**Figure 2:**
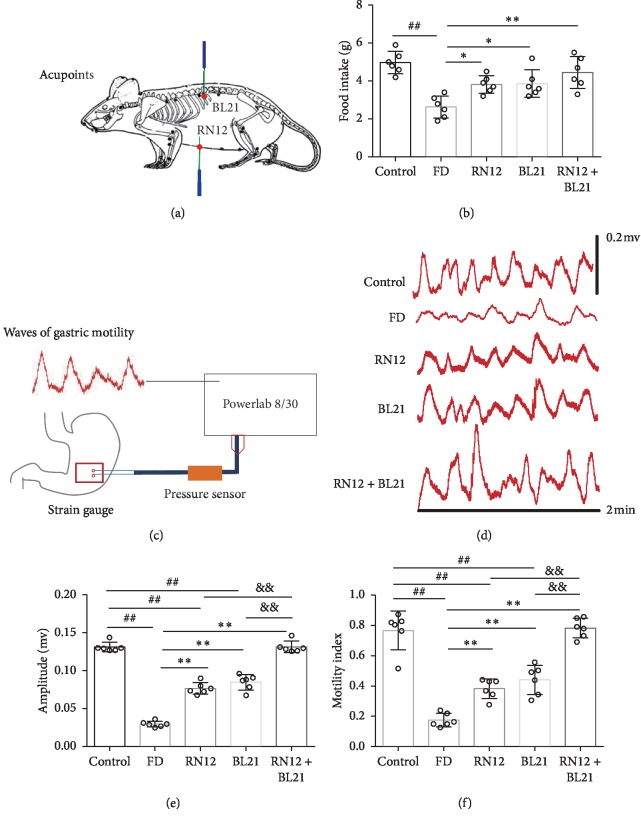
EA increases the food intake and gastric motility of FD rats. (a) The location of RN12 and BL21. (b) Food intake, *n* = 6. (c–f) Gastric motility. (c) Recordings of gastric motility. (d) Representative waves of gastric motility. (e, f) Gastric motility amplitude and gastric motility index, *n* = 6. Compared with the control group, ^##^*P* < 0.01. Compared with the FD group, ^*∗∗*^*P* < 0.01, ^*∗*^*P* < 0.05. Compared with the RN12 or BL21 group, ^&&^*P* < 0.01.

In order to assess the effect of EA at RN12 and BL21 on gastric motility, we chose FD rats as the research object and recorded the gastric motility and food intake of the FD rats upon EA at RN12 and BL21. The location of RN12 and BL21 is shown in Figure 2(a). The food intake data showed that the food intake of the FD group was significantly decreased compared to that of the control group, and EA at RN12, BL21, and RN12 + BL21 increased the food intake of the FD rats (Figure 2(b)). The gastric motility data showed that the gastric motility amplitude and gastric motility index were severely decreased in the FD, RN12, and BL21 groups compared with the control group. Compared with the FD group, EA at RN12, BL21, and RN12 + BL21 strongly increased the amplitude and motility index. Furthermore, compared with the RN12 and BL21 group, the RN12 + BL21 group exhibited significantly increased amplitude and motility index (Figures 2(c)–2(f)). Collectively, these data demonstrated that EA at RN12 and BL21 enhances food intake and gastric motility in FD rats and that the compatibility of RN12 and BL21 had an additional effect.

### 3.2. The Effect of EA at RN12 and BL21 on Hippocampus Glutamate

**Figure 3:**
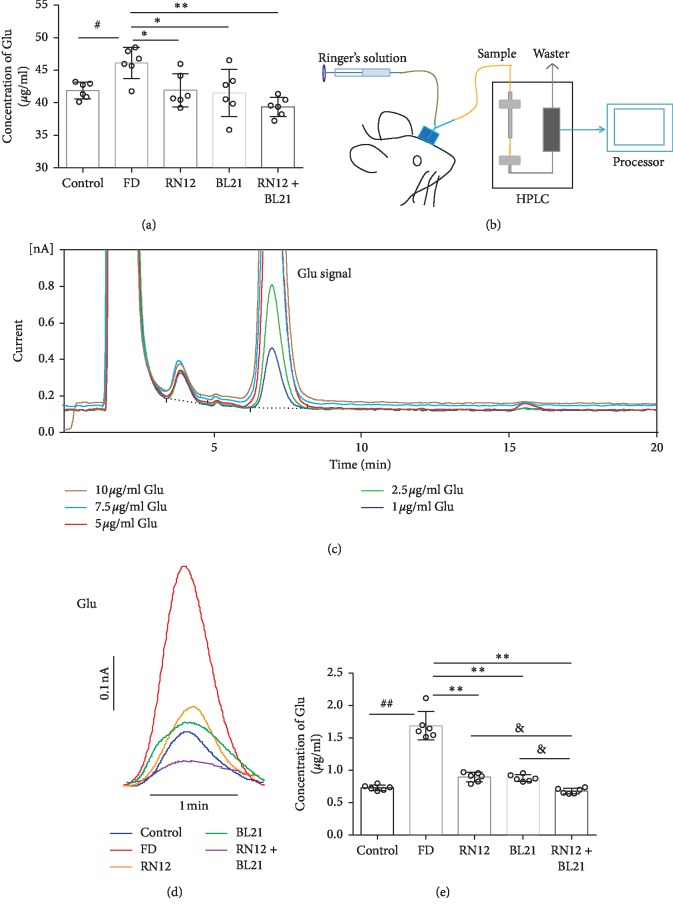
EA reduces the glutamate content in the hippocampus of FD rats. (a) The quantification of glutamate in the hippocampus by colorimetric assay. *n* = 6. Compared with the control group, ^#^*P* < 0.05. Compared with the FD group, ^*∗∗*^*P* < 0.01, ^*∗*^*P* < 0.05. (b–e) Microdialysis combined with HPLC. (b) The microdialysis combined with the HPLC procedure. (c) Representative curves of glutamate standards. (d) Representative curves of extracellular glutamate in the hippocampus. (e) Quantification of glutamate, *n* = 6. Compared with the control group, ^##^*P* < 0.01. Compared with the FD group, ^*∗∗*^*P* < 0.01. Compared with the RN12 and BL21 groups, ^&^*P* < 0.05.

The hippocampus plays a critical role in the regulation of gastric motility by EA at BL21 and RN12, and central glutamate is closely related to gastric motility. Among the pathological processes involved in FD, gastric motility dysfunction is important. In this study, we evaluated whether hippocampal glutamate is involved in the improvement of FD by EA at BL21 and RN12. We detected glutamate content in the hippocampus by a colorimetric assay and microdialysis combined with HPLC. As shown by the colorimetric assay results, hippocampal glutamate content was increased in the FD group compared with the control group, and compared with that in the FD group, glutamate content was significantly decreased in the three EA groups (RN12, BL21, and RN12 + BL21) (Figure 3(a)). Moreover, the results of microdialysis combined with HPLC showed that the extracellular glutamate content in the hippocampus increased significantly in the FD group compared with the control group, and compared with the FD group, the EA groups (RN12, BL21, and RN12 + BL21) exhibited decreased the glutamate content; furthermore, the decrease in glutamate content was greater in the RN12 + BL21 group than in the RN12 and BL21 groups (Figures 3(b)–3(e)). These results demonstrated that FD increased the content of glutamate in the hippocampus whereas EA reduces this content.

### 3.3. The Effect of EA at RN12 and BL21 on the Expression of NR1, nNOS, and sGC in the Hippocampus

**Figure 4:**
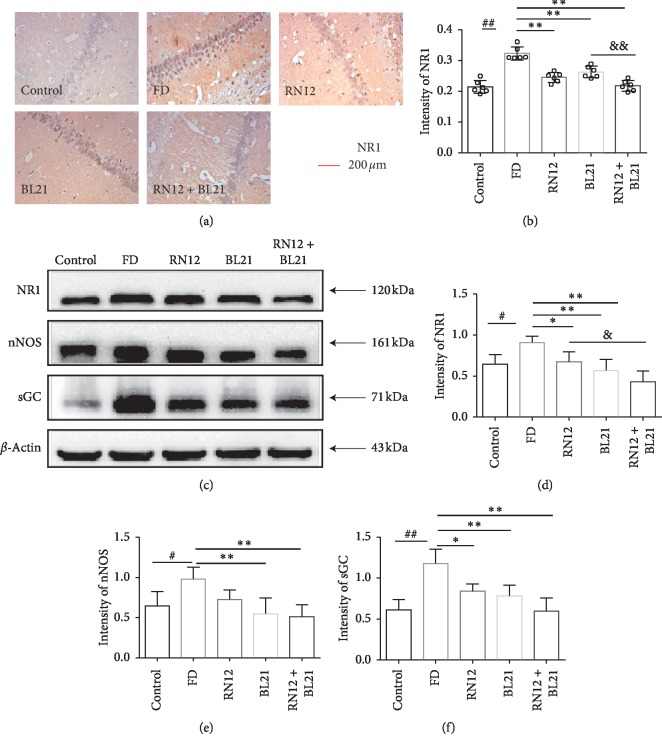
EA suppresses the expression of NR1, nNOS, and sGC in the hippocampus of FD rats. (a, b) Immunohistochemistry. (a) Representative photomicrographs of NR1 in the hippocampus of each group, scale bar, 200 *μ*m. (b) Quantification of NR1 expression, *n* = 6. Compared with the control group, ^##^*P* < 0.01. Compared with the FD group, ^*∗∗*^*P* < 0.01. Compared with the BL21 group, ^&&^*P* < 0.01. (c–f) Western blot analysis. (c) Representative bands for NR1, nNOS, and sGC (top); the representative band for *β*-actin as the internal control (bottom). (d–f) Quantification of NR1, nNOS, and sGC expression in the hippocampus, *n* = 3. Compared with the control group, ^##^*P* < 0.01, ^#^*P* < 0.05. Compared with the FD group, ^*∗∗*^*P* < 0.01, ^*∗*^*P* < 0.05. Compared with the RN12 group, ^&^*P* < 0.05.

NMDAR is widely distributed in the hippocampus and plays an important role in regulating gastric motility. NMDAR subunit 1 (NR1) is crucial for the function of the ion channel of NMDAR. The activation of NMDAR may cause NO release and cGMP synthesis. Based on previous reports, we investigated whether NR1, NO, or cGMP is involved in the improvement of FD by EA at RN12 and BL21. We initially determined the expression of NR1 in the hippocampus by immunohistochemistry. Compared with that in the control group, the expression of NR1 in the FD group was increased, and compared with that in the FD group, the expression of NR1 in the EA groups (RN12, BL21, and RN12 + BL21) was significantly decreased. Moreover, the decrease in the expression of NR1 in the RN12 + BL21 group was greater than that in the BL21 group, which suggests that EA, especially EA at RN12 + BL21, can inhibit the expression of NR1 in FD rats (Figures 4(a) and 4(b)). Furthermore, we determined the expression of NR1, nNOS (NO synthase), and sGC (cGMP synthase) in the hippocampus by Western blotting. Compared with that in the control group, the expression of NR1, nNOS, and sGC was significantly increased in the FD group, and compared with that in the FD group, the expression of NR1, nNOS, and sGC was strongly decreased in the RN12, BL21, and RN12 + BL21 groups. The results revealed that FD increased the expression of NR1, nNOS, and sGC in the hippocampus, whereas EA decreased this response (Figures 4(c)–4(f)).

### 3.4. The Effect of EA at RN12 + BL21 on Gastric Motility after Microinjection of MK-801, L-NAME, or MB into the Hippocampus

**Figure 5:**
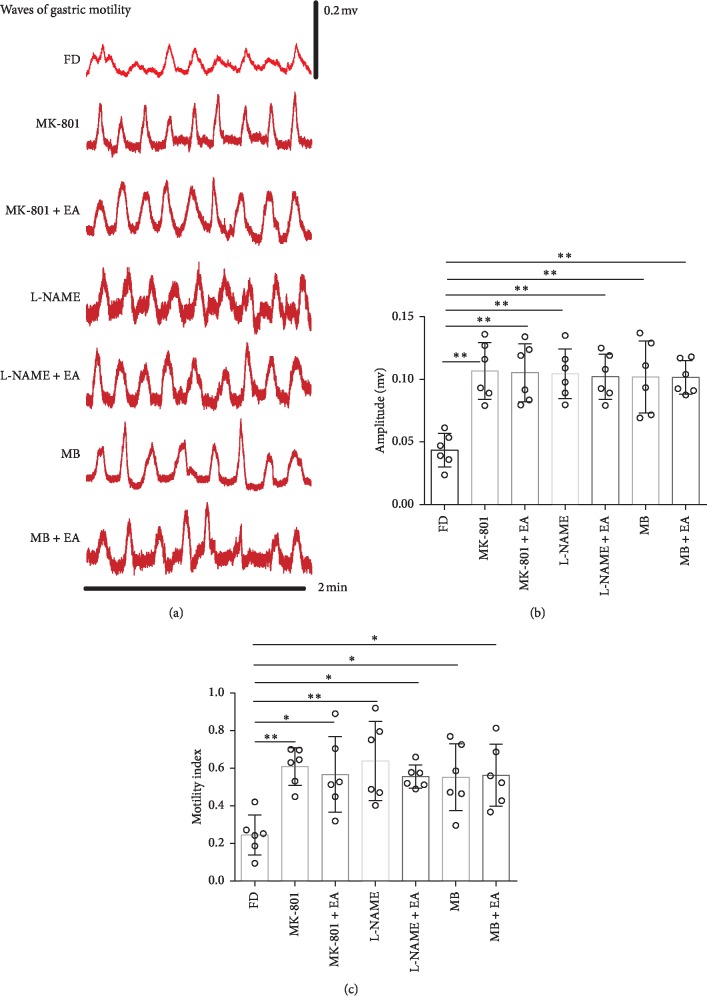
NMDAR, nNOS, and sGC are critical for the EA-induced enhancement of gastric motility. (a–c) Gastric motility. (a) Representative waves of gastric motility. (b, c) Gastric motility amplitude and gastric motility index, *n* = 6. Compared with the FD group, ^*∗∗*^*P* < 0.01, ^*∗*^*P* < 0.05.

To further investigate whether the regulation of gastric motility in FD rats by EA is dependent on the NMDAR-NO-cGMP pathway, MK-801, L-NAME, or MB was injected into the bilateral hippocampi of FD rats to block NMDAR, nNOS, or sGC, respectively, and gastric motility was recorded with a gastrointestinal pressure sensor. The results revealed that microinjection of NMDA receptor antagonist MK-801 into hippocampi restored gastric motility of FD rats, application of nNOS and sGC antagonists repeated the effect of MK-801 on gastric motility, and EA had no further effects on gastric motility in the presence of these antagonists in FD rats, indicating that NMDAR, NO, and cGMP play a critical role in the regulation of gastric motility by EA at RN12 + BL21 (Figures 5(a)–5(c)).

## 4. Discussion

The compatibility of acupoints is one of the key factors affecting the clinical efficacy of acupuncture. According to traditional Chinese medicine (TCM), the compatibility of the back-shu and the front-mu points, which is based on the theories of qi jie and yin-yang, has a good clinical effect. BL21 is the gastric back-shu point, while RN12 is the gastric front-mu point. Acupuncture at BL21 and RN12 can treat gastrointestinal disease. Among the pathological processes involved in gastrointestinal disease, gastrointestinal motility dysfunction, including reduced gastrointestinal motility, is important [28]. Acupuncture may have regulatory effects on gastrointestinal motility. One study found that electroacupuncture (EA) at ST36 promotes gastric motility of FD rats [29]. Research on rats with diabetic gastroparesis has indicated that EA at SP6, ST36, and ST21 increases gastrointestinal motility [30]. Moreover, some clinical studies have demonstrated that acupuncture at Siguan acupoints inhibits the excessive gastrointestinal motility induced by mosapride citrate and promotes the suppression of gastrointestinal motility induced by loperamide [31]. Therefore, we can see that acupuncture has a dual regulatory effect on gastrointestinal motility. Here, we demonstrated that EA at RN12 + BL21, compared with EA at RN12 or BL21, enhances decreased gastric motility in functional dyspepsia (FD), and RN12 + BL21 showed a greater effect on gastric motility, suggesting that the compatibility of the acupoints had an additional effect. Although many studies have investigated the mechanism of acupuncture, the mechanism underlying such an additional effect remains unclear.

Neurotransmitters, modulators, and pathways of the central nervous system are important factors in the regulation of gastrointestinal motility by EA [32]. The study showed that both GABA and glutamate in the brainstem circuit are involved in the regulation of gastric motility by EA at ST36 [33]. Glutamate is the major excitatory neurotransmitter in the central nervous system and its receptors include both ionotropic receptors and metabotropic receptors. Previous studies have suggested that glutamate regulates gastric motility through specific NMDA receptor activity, but not non-NMDA receptor activity [34]. For example, microinjection of glutamate into the nucleus ambiguus (NA) partially inhibits gastric motility through the activation of the NMDAR-NO pathway, and microinjection of glutamate into the hippocampus inhibits gastric motility through NMDAR [35, 36]. Moreover, one study showed that acute high-fat diet upregulated glutamatergic signalling in the DMV, thus increasing gastric motility [17]. These studies indicated that the change of the glutamatergic system could affect gastric motility. Meanwhile, studies have shown that NMDAR antagonists are potential clinical targets for the treatment of FD [37]. Interestingly, in our study, we found that FD rats were followed by gastric motility disfunction and also followed by increased hippocampal glutamate and the upregulated NMDAR-NO-cGMP pathway, however, when microinjection of the NMDAR, nNOS, or sGC inhibitor into the hippocampus could restore the gastric motility of FD rats. These results suggest that the decreased gastric motility of FD rats is associated with the overexpression of the hippocampal glutamatergic system. However, whether EA enhances gastric motility of FD rats involving the hippocampal glutamatergic system remains to be elucidated.

Studies have revealed that EA can affect the hippocampus, as supported by imaging and the expression of *c-fos*, and has also shown that the hippocampus is involved in EA regulation of the gastrointestinal motility [38, 39]. Our study found that the hippocampal glutamatergic system participated in the regulation of gastric motility. To examine whether EA at RN12 and BL21 relates to the hippocampal glutamatergic system, we examined the concentration of glutamate and the expression of NR1, nNOS, and sGC in the hippocampus. The results showed that EA could inhibit hippocampal glutamatergic system of FD rats. To further study the involvement of NMDAR-NO-cGMP pathway in the regulation of gastric motility by EA, we performed EA at RN12 + BL21 along with the injection of NMDAR, nNOS, or sGC inhibitor, respectively, into the hippocampus. We found that EA had no effects on gastric motility in the presence of inhibitor, suggesting that NMDAR-NO-cGMP pathway participated in the regulation of gastric motility by EA. In conclusion, these observations suggest that the EA regulated gastric motility of FD rats might be related to the suppression of glutamate and the NMDAR-NO-cGMP pathway. Why does the inhibition of the glutamatergic system in the hippocampus lead to the enhancement of gastric motility? Firstly, it may be related to the vagus nerve activation or the sympathetic nerve inhibition [40]. Secondly, the overexpression of the glutamatergic system can lead to the release of a large amount of NO, which can induce hippocampal neuronal damage and death and is suppressed to restore gastric motility [41]. Thirdly, studies have indicated that the NO-cGMP pathway plays a critical role in smooth muscle relaxation, so suppressing this pathway may promote gastric smooth muscle contraction [42]. Therefore, we speculate that there may be complex mechanisms underlying the glutamatergic system in the central nervous system to regulate gastric motility, and these mechanisms remain to be explored in future experiments.

In summary, our findings revealed that EA at RN12 and BL21 enhanced gastric motility in FD rats and that EA at a combination of RN12 and BL21 had an additional effect. The decreased gastric motility of FD rats was associated with the overexpression of the hippocampal glutamatergic system, and EA at RN12 and BL21 could reduce glutamate and suppress the NMDAR-NO-cGMP pathway in the hippocampus of FD rats. Furthermore, we found that EA had no effects on gastric motility in the presence of NR1, nNOS, and sGC inhibitors. Therefore, we propose that the glutamatergic system is involved in the hippocampus of EA at RN12 and BL21 that regulates gastric motility.

## Data Availability

The data used to support the findings of this study are available from the corresponding author upon request.
